# Diversity distribution characteristics and influencing factors of understory herbaceous in *Poplar* plantations of different ages in the LuXi Yellow floodplain

**DOI:** 10.3389/fpls.2026.1830354

**Published:** 2026-07-30

**Authors:** Zhibao Wang, Linlin Sun, Jing Liang, Haibing Wu, Shouchao Yu, Xiangbin Gao, Qiandong Qiu, Yingchang Xiu, Hongxia Zhao, Jiacai Tu

**Affiliations:** 1 College of Agriculture and Biology, Liaocheng University, Liaocheng, China; 2 School of Life Sciences, Shandong University, Qingdao, China; 3 Soil Research Institute, Shanghai Academy of Landscape Architecture Science and Planning, Shanghai, China

**Keywords:** age, plant diversity, *Poplar* plantations, soil physicochemical properties, Yellow floodplain

## Abstract

To explore the distribution characteristics of plant diversity and its relationships with environmental factors under poplar plantations of different ages in the Luxi Yellow River Floodplain, and to reveal the maintenance mechanisms of plant diversity in planted forests, four poplar plantations with different stand ages (10 y, 30 y, 40 y, 50 y) and a grassland used as the control were selected in Jiucheng Forest Farm, Gaotang County, Shandong Province. Field investigations combined with laboratory analyses were adopted to systematically examine the plant community characteristics and environmental factors under poplar stands of different ages. A total of 21 plant species were recorded in all quadrats, belonging to 9 families and 21 genera. *Setaria viridis*, *Phragmites australis*, and *Erigeron canadensis* were recognized as the dominant species. The highest species rich (12 species) was observed in the control grassland, which was significantly higher than those recorded in all stand ages. Compared with the control grassland, the Shannon-Wiener index and Simpson index of herbaceous plants under poplar plantations of different ages were found to increase initially and then decrease with increasing stand age. The Pielou index reached its maximum value at the stand age of 10 years and decreased gradually thereafter. The Margalef index exhibited slight overall fluctuations and declined slowly after reaching its peak at the standing age of 30 years. No significant differences were detected in soil pH, electrical conductivity (EC), and total nitrogen (TN) among different stand ages. Soil bulk density (SBD) and total phosphorus content (TP) were observed to decrease with increasing stand age, and the minimum values (1.28 g·cm^-^³ and 0.45 g·kg^-^¹) were obtained at the stand age of 50 years. Soil water content (SWC), total porosity (STP), soil organic carbon (SOC), available potassium (AK), and TN were found to increase with stand age, and the maximum values (6.36%, 54.68%, 9.44 g·kg^-^¹, 93.67 mg·kg^-^¹, and 0.54 g·kg^-^¹, respectively) were reached at the stand age of 50 years. Results obtained from correlation analysis, multiple regression analysis, demonstrated that canopy closure (CD), stand density (SD), SWC, and SOC were identified as the key environmental factors regulating variations in plant diversity of poplar plantations in the Luxi Yellow River Floodplain.

## 1 Introduction

Understory vegetation acts as an essential component of forest ecosystems and delivers irreplaceable ecological benefits in biodiversity maintenance (Chen and Cao, 2014), soil and water conservation (Wang et al., 2024), climatic regulation (Stickley and Fraterrigo, 2021), and ecological environment improvement (Mcalpine et al., 2021). Large-scale afforestation programs have been implemented nationwide in China for ecological restoration, and substantial plant resources have gradually developed in the understory layers of artificial forest lands. Understory vegetation constitutes a vital functional component of plantation ecosystems and plays a pivotal role in maintaining the structural integrity and functional stability of overall forest communities (Liu et al., 2022). Plant diversity has long been a core research topic in biodiversity and ecological studies. Plant growth and spatial distribution are comprehensively influenced by multiple environmental variables including geographical conditions, forest stand types and soil properties (Yao et al., 2023), which leads to high complexity in the driving mechanisms of plant community variation. In this context, systematic exploration of the interactive relationships between understory plant community traits and environmental factors, as well as identification of the dominant environmental drivers of plant diversity in artificial plantations, can effectively support the stability maintenance and sustainable development of forest ecosystems (Li et al., 2022).

Previous studies have demonstrated that understory plant diversity is affected by intricate interactive factors rather than a single environmental variable, which is closely associated with local ecological conditions and community structure. Existing studies have verified that understory plant diversity in plantations is commonly regulated by stand structure (Helbach et al., 2022), stand age (Lee et al., 2024), stand density (Matsushita et al., 2025) and growth stage (Han et al., 2021). Multiple studies have confirmed that the complexity and density of stand structure indirectly drive the variation of understory plant diversity in temperate European plantations by modulating understory light availability and soil moisture (Helbach et al., 2022; Zhao et al., 2023; Wu et al., 2025). Several studies have reported that understory plant species richness increases continuously with the growth of stand age. For instance, the 12-year-old *Phellodendron chinense* plantation possesses more understory plant species (30 species) than the 2-year-old plantation, with the highest Shannon-Wiener index and Simpson index values (Huang et al., 2026). Similarly, mature natural *Picea purpurea* forests have much higher Shannon-Wiener index (2.66) and Simpson index (0.89) compared with young stands (0.56 and 0.23), indicating that the optimized understory microhabitat formed with increasing stand age can provide suitable habitats for more plant species (Su et al., 2022). Increasing stand age can also trigger functional turnover of plant community composition. Specifically, herbaceous communities tend to transform into shade-tolerant and drought-resistant species communities along with the rise of stand density and stand age in *Robinia pseudoacacia* forests (Zhao et al., 2023). In *Caragana korshinskii* sand-fixation forests, herbaceous communities undergo obvious succession from *Corispermum* and *Agriophyllum* communities to *Stipa breviflora* communities as stand age increases (Yu et al., 2024). In addition, close correlations exist between understory plants and soil properties across plant communities of different stand ages. Species quantity in *Phellodendron chinense* stands is mainly regulated by soil phosphorus potassium calcium and magnesium contents (Xie et al., 2025). The Margalef index in *Picea purpurea* forests is largely affected by soil total nitrogen organic carbon and total phosphorus and all these soil indicators present higher values in mature stands than in young stands (Tang et al., 2025). For *Cunninghamia lanceolata* plantations mature stands have distinctly higher contents of soil organic carbon total nitrogen and total phosphorus than middle aged stands and soil porosity and water holding capacity also get improved with the extension of stand age (Huang et al., 2026). Su et al. (2022) adopted structural equation modeling and verified that soil nutrient availability rather than light heterogeneity or substrate diversity acts as the core factor determining understory plant diversity in subtropical forests. The interaction between stand age and soil organic matter exerts remarkable influences on understory herb diversity in *Poplar* plantations of northern China (Lee et al., 2024). Such coupling effects of stand age and soil characteristics including soil moisture and nitrogen content jointly shape the distribution pattern of understory plant diversity in *Robinia pseudoacacia* plantations on the Loess Plateau meanwhile plantation cultivation can effectively alter soil physical attributes (Wu et al., 2021; Guo et al., 2021; Tan et al., 2024). In the research on coniferous plantations Matsushita et al. (2025) comprehensively evaluated the relative contributions of soil conditions stand structure and landscape factors to understory plant communities and offered methodological references for multi scale ecological analysis. All these previous findings lay solid theoretical foundations for systematic investigations on understory plant diversity within artificial forest ecosystems.

The Luxi Yellow River Floodplain represents a typical ecologically vulnerable zone in China. This unique landform develops from long term fluvial water sediment interactions of the Yellow River coupled with continuous human disturbance. Its distinctive geographic setting, special formation processes and prominent ecological problems make the region an ideal study area to reveal the geographic variation and ecological succession patterns across the Yellow River Basin. This floodplain region suffers from multiple ecological constraints. Common environmental problems include progressive soil deterioration derived from soil erosion, salinization and nutrient depletion, simplified and unstable forest landscapes with reduced biological diversity and weakened shelterbelt ecological capacity, as well as groundwater depletion induced by over exploitation. Improving the health and stability of artificial forest communities, clarifying the diversity characteristics of understory plant assemblages in plantation stands, and realizing sustainable water resource management constitute the primary targets of local ecological remediation. Relevant research findings can provide practical theoretical support for ecological restoration and ecosystem rehabilitation in alluvial plain areas.

*Poplar* plantation construction serves as a key ecological restoration approach for environmental improvement in the Yellow River Floodplain, and such artificial forests have been extensively cultivated throughout the region (Chen et al., 2022). Populus trees are widely applied for afforestation practices across northern China due to their superior adaptive traits including drought resistance, tolerance to barren soil conditions, fast growth rates and strong environmental plasticity. These tree species play essential roles in windbreak and sand fixation as well as forest ecosystem restoration, making them the primary shelterbelt tree species for ecological construction in the Yellow River Floodplain (Duan et al., 2022). Existing academic research still lacks sufficient systematic exploration of understory plant diversity and its coupling relationships with environmental variables in regional *Poplar* plantations. It is therefore necessary to carry out in depth targeted investigations in the Yellow River Floodplain. This study selected *Poplar* plantations with different stand ages at Jiucheng Forest Farm of Gaotang County in Shandong Province as the research materials. Field investigations were conducted to collect data on understory plant community structures and soil physicochemical properties. Correlation analysis and multiple regression analysis were applied to explore the distribution characteristics of understory plant diversity and soil environmental indicators, and to clarify their internal interaction relationships. This paper further elucidates the influencing mechanisms of environmental factors on the spatial variation of understory plant diversity. The research results can provide reliable scientific references for regional plant diversity protection and standardized sustainable management of forest ecosystems in the Luxi Yellow River Floodplain.

## 2 Materials and methods

### 2.1 Study area

**Figure 1:**
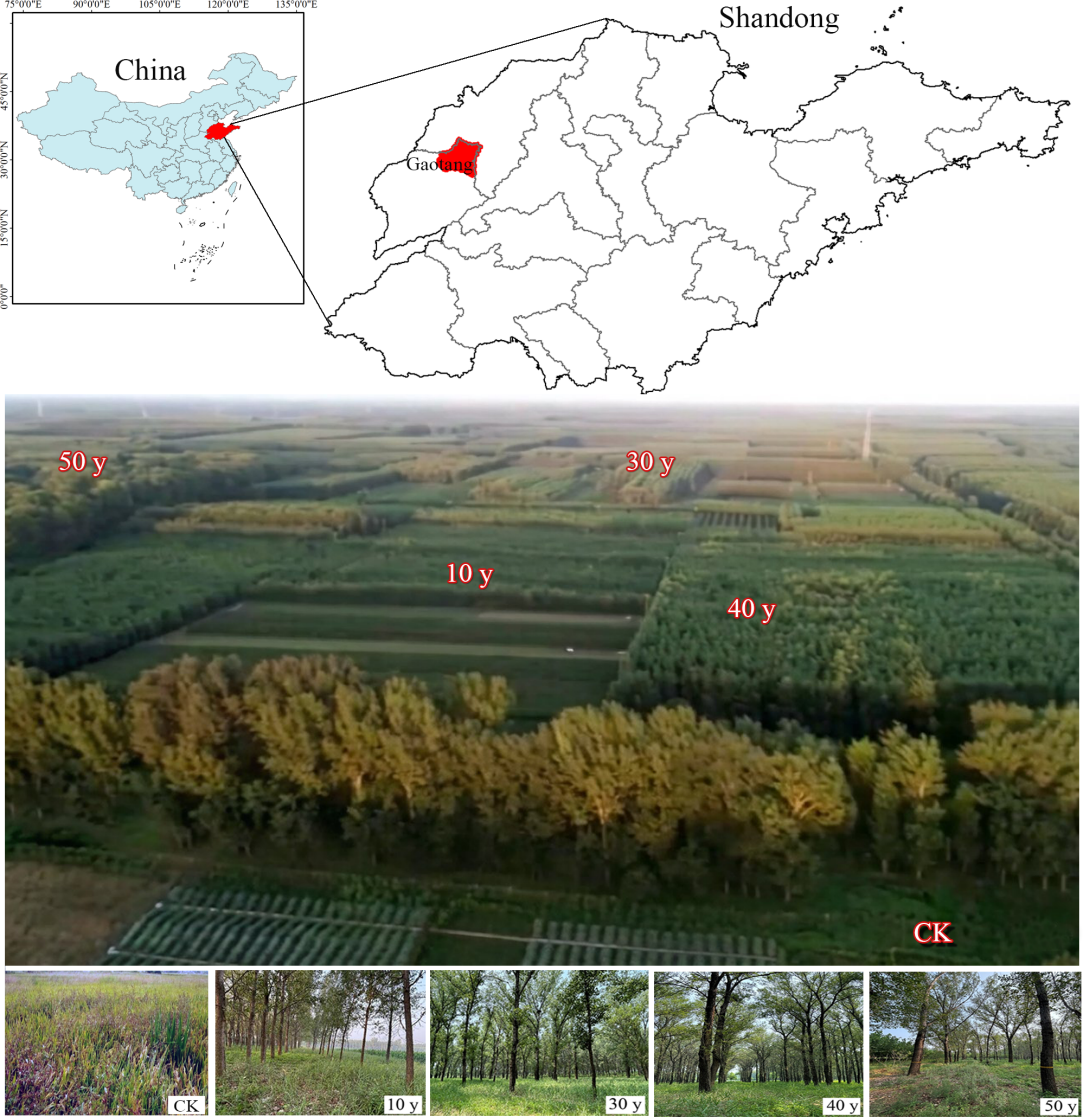
Study area.

The study area was located in Jiucheng Forest Farm, Qingping Town, Gaotang County, Shandong Province (35°47′~38°03′ N, 114°16′~119°32′ E), which belongs to the Luxi Yellow River Floodplain (**Figure 1**). This area is characterized by a warm temperate monsoon climate. The annual average temperature ranges from 12 to 13 °C, the annual average precipitation varies from 446.5 to 916.0 mm, the annual average humidity is approximately 75%, the annual sunshine duration ranges from 2463 to 2741 hours, and the frost-free period is about 200 days. The soil type is identified as sandy loam. The vegetation is classified as warm temperate deciduous broad-leaved forest. The dominant woody species are mainly *Populus euramericana*, *Populus tomentosa*, *Robinia pseudoacacia* and *Sabina chinensis*. The main herbaceous species include *Setaria viridis*, *Phragmites australis*, *Erigeron canadensis*, *Imperata cylindrica*, *Sonchus oleraceus* and other associated species.

### 2.2 Plot establishment and vegetation investigation methods

In July 2023, a general field survey was carried out in Jiucheng Forest Farm of Gaotang County to investigate *Poplar* plantations with varied stand ages. *Poplar* stands aged 10, 30, 40 and 50 years with consistent stand conditions were selected as research subjects as shown in **Table 1**, and natural grasslands free from *Poplar* plantation disturbance were set as the control group. Three standard plots were established in grassland areas with an interval over 20 meters between adjacent plots. Three 1 m x 1 m quadrats were arranged within each grassland standard plot to survey herbaceous plant diversity, forming a total of nine monitoring quadrats for grassland investigation. Three replicated standard plots were arranged for *Poplar* plantations of each age class, and twelve standard plots were established for the four age groups in total. Three 20 m x 20 m arbor monitoring quadrats were further laid out in each standard plot of *Poplar* plantations with intervals exceeding 20 meters, and the total number of such quadrats reached 36. All individual trees within each arbor quadrat were measured in detail. Stand related indicators including canopy density, stand density, mean tree height, diameter at breast height and crown width were recorded. The latitude and longitude information of each sampling plot was recorded using a handheld global positioning system device.

**Table 1:** Overview of sample plots.

Age/y	CD	SD/(tree·hm^-2^)	MTH/m	DBH/cm	W/m
CK	—	—	—	—	—
10	0.21 ± 0.02d	916.67 ± 52.04c	21.33 ± 0.58d	24.17 ± 0.76c	6.12 ± 0.31c
30	0.46 ± 0.05c	450.00 ± 25.00c	32.67 ± 0.76c	36.00 ± 1.00b	13.26 ± 0.30b
40	0.65 ± 0.03b	391.67 ± 14.43b	35.00 ± 1.00b	37.67 ± 0.76b	13.93 ± 1.10b
50	0.77 ± 0.06a	341.67 ± 14.43a	37.83 ± 1.04a	39.83 ± 1.04a	17.07 ± 1.69a

CD, canopy closure; SD, stand density; MTH, mean tree height; DBH, diameter at breast height; W, crown breadth. Different lowercase letters in the same column indicate significant differences at the 0.05 probability level. The same applies to the following tables.

Three herb quadrats of 1 m × 1 m were arranged in a triangular pattern within each standard plot. The species name, individual number, height, and coverage of herbaceous plants in each quadrat were recorded in detail.

### 2.3 Soil collection and index determination

Soil sampling was conducted using the ring knife method. Three 1 m×1 m soil sampling points were set along the diagonal of each 20 m×20 m standard plot. After removing surface litter, a 100 cm³ ring knife was inserted into the 0–20 cm soil layer at each sampling point, and the ring knife was wrapped with plastic wrap immediately after being taken out. Meanwhile, soil samples were dug with a shovel at each sampling point, placed into self-sealing bags, and transported back to the laboratory together with the ring knives. All samples were stored in a refrigerator at 4°C for preservation. In the laboratory, the fresh weight of the soil was measured first, and then the soil was dried to a constant weight in an oven at a constant temperature of 80°C. The dry weight was measured with a precision of 0.0001 g, and the soil water content (SWC) was determined by the oven-drying method. Soil physical properties such as soil bulk density (SBD), field capacity (FC), and soil total porosity (STP) were measured using the ring knife method. After the air-dried soil was ground and passed through a 100-mesh sieve, the soil organic carbon content was determined by the potassium dichromate external heating method, the nitrogen content was measured by the Kjeldahl method, the phosphorus content was determined by the molybdenum-antimony anti-colorimetric method, and the soil available potassium (AK) content was measured by the flame photometric method (Bao, 2000). The soil pH value was determined using a pH meter, and the electrical conductivity (EC) was measured with an electrical conductivity meter.

### 2.4 Calculation of herbaceous plant diversity

Based on the field investigation data of vegetation, the important value (*P_i_*), Shannon-Wiener index (*H*), Simpson index (*H’*), Pielou index (*J*), and Margalef index (*M*) of herbaceous species were calculated. The corresponding formulas are given as follows. The important value is calculated by Equations (Shannon, 1948; Simpson, 1949; Curtis and McIntosh, 1950; Pielou, 1996):

(1) $$P_{i}=\left(\text{Relative coverage}+\text{Relative density}+\text{Relative frequency}+\text{Relative height}\right)/4$$

(2) $$H=—\sum_{i=1}^{S}P_{i}\ln P_{i}$$

(3) $$H^{\prime }=\sum_{i=1}^{S}P_{i}^{2}$$

(4) $$J=\frac{H}{\ln S}$$

(5) M=(S−1)/lnN

In these equations, *S* stands for the number of species in each quadrat, *N* represents the total number of individual plants in each quadrat, and *N_i_* denotes the number of individuals of the *i*-th species.

### 2.5 Data processing

Data collation and statistical analysis were performed using Microsoft Excel 2010 and SPSS 26.0 software. Prior to formal data analysis, homogeneity of variance test was conducted for all collected datasets. Logarithmic transformation was applied to datasets when heterogeneous variance was detected. One-way analysis of variance (One-way ANOVA) and least significant difference (LSD) method were adopted to analyze the differences in herbaceous plant diversity indices and soil physicochemical properties under *Poplar* plantations with different stand ages. The significance level for all statistical tests was set at α ≤ 0.05. Pearson correlation analysis was used to examine the correlation between environmental factors and plant diversity indices. Multiple regression equation was established to identify the key factors influencing plant diversity. All analytical figures in this study were generated using Origin Pro 9.0 software.

## 3 Results

### 3.1 Plant composition under *Poplar* plantations of different stand ages

A total of 21 plant species, belonging to 9 families and 21 genera, were recorded in all investigated quadrats (**Table 2**). Fourteen herbaceous species within 14 genera and 7 families were discovered in the control grassland. The dominant species were *Phragmites australis*, *Erigeron canadensis* and *Setaria viridis*, with corresponding important values of 25.57%, 14.18% and 13.04%, respectively (Equation 1). Eleven herbaceous species across 11 genera and 8 families were found in the 10-year-old stand, and the dominant species were *Phragmites australis* (26.73%), *Setaria viridis* (14.90%) and *Medicago lupulina* (9.81%). Ten herbaceous species belonging to 10 genera and 7 families were observed in the 30-year-old stand, with *Phragmites australis* (25.20%), *Hemerocallis minor* (11.75%) and *Erigeron canadensis* (11.69%) as the dominant species. Nine herbaceous species within 9 genera and 7 families were detected in the 40-year-old stand, and the dominant species were *Phragmites australis* (24.65%), *Hemerocallis minor* (14.23%) and *Medicago lupulina* (11.96%). Eight herbaceous species across 8 genera and 7 families were identified in the 50-year-old stand, with *Phragmites australis* (21.76%), *Eriophorum vaginatum* (16.99%) and *Medicago lupulina* (15.85%) as the dominant species. The results indicated that *Phragmites australis* (Poaceae), *Humulus scandens* (Cannabaceae), *Rubia cordifolia* (Rubiaceae) and *Chromolaena odorata* (Asteraceae) were distributed in all quadrats under *Poplar* plantations of different stand ages.

**Table 2:** Dominant species and important values of understory plants at different forest ages.

Species				Importance value/%				
Family	Genera	Species	Life form	CK	10 y	30 y	40 y	50 y
Poaceae	*Phragmites*	*Phragmites australis*	B	25.57	26.73	25.20	24.65	21.76
Poaceae	*Setaria*	*Setaria viridis*	A	13.04	14.9	—	—	—
Asteraceae	*Erigeron*	*Erigeron canadensis*	A	14.18	9.10	11.69	—	—
Cannabaceae	*Humulus*	*Humulus scandens*	B	4.54	6.92	9.17	6.48	11.01
Cyperaceae	*Cyperus*	*Cyperus iria*	A	3.86	—	—	—	—
Poaceae	*Digitaria*	*Digitaria sanguinalis*	B	4.91	—	—	—	—
Asteraceae	*Artemisia*	*Artemisia caruifolia*	A	3.73	—	—	—	—
Poaceae	Imperata	*Imperata cylindrica*	B	5.08	—	—	—	—
Amaranthaceae	*Kochia prostrata*	*Kochia scoparia*	A	5.86	—	—	—	—
Rubiaceae	*Rubia*	*Rubia cordifolia*	B	5.24	5.75	7.26	10.20	10.13
Cyperaceae	*Eriophorum*	*Eriophorum scheuchzeri*	B	4.12	6.02	—	10.75	16.99
Boraginaceae	*Trigonotis*	*Trigonotis peduncularis*	A	3.14	—	—	—	—
Poaceae	*Echinochloa*	*Echinochloa crus-galli*	B	4.49	—	—	—	—
Asteraceae	*Eupatorium*	*Chromolaena odorata*	B	2.25	3.48	3.64	4.36	3.55
Liliaceae	*Hemerocallis*	*Hemerocallis minor*	A	—	7.67	11.75	14.23	—
Leguminosae	*Medicago*	*Medicago lupulina*	B	—	9.81	11.15	11.96	15.85
Amaranthaceae	*Salsola*	*Salsola collina*	B	—	5.33	—	—	10.74
Amaranthaceae	*Chenopodium*	*Chenopodium album*	A	—	4.31	7.86	——	9.96
Asteraceae	*Conyza*	*Erigeron bonariensis*	A	—	—	5.94	6.74	—
Leguminosae	*Vicia*	*Vicia sepium*	B	—	—	6.35	—	—
Asteraceae	*Sonchus*	*Sonchus oleraceus*	A	—	—	—	10.63	—

A represents annual species, and B represents perennial species. The symbol “—” indicates that the species is absent in the corresponding stand age.

### 3.2 Plant diversity in *Poplar* plantations of different stand ages

**Figure 2:**
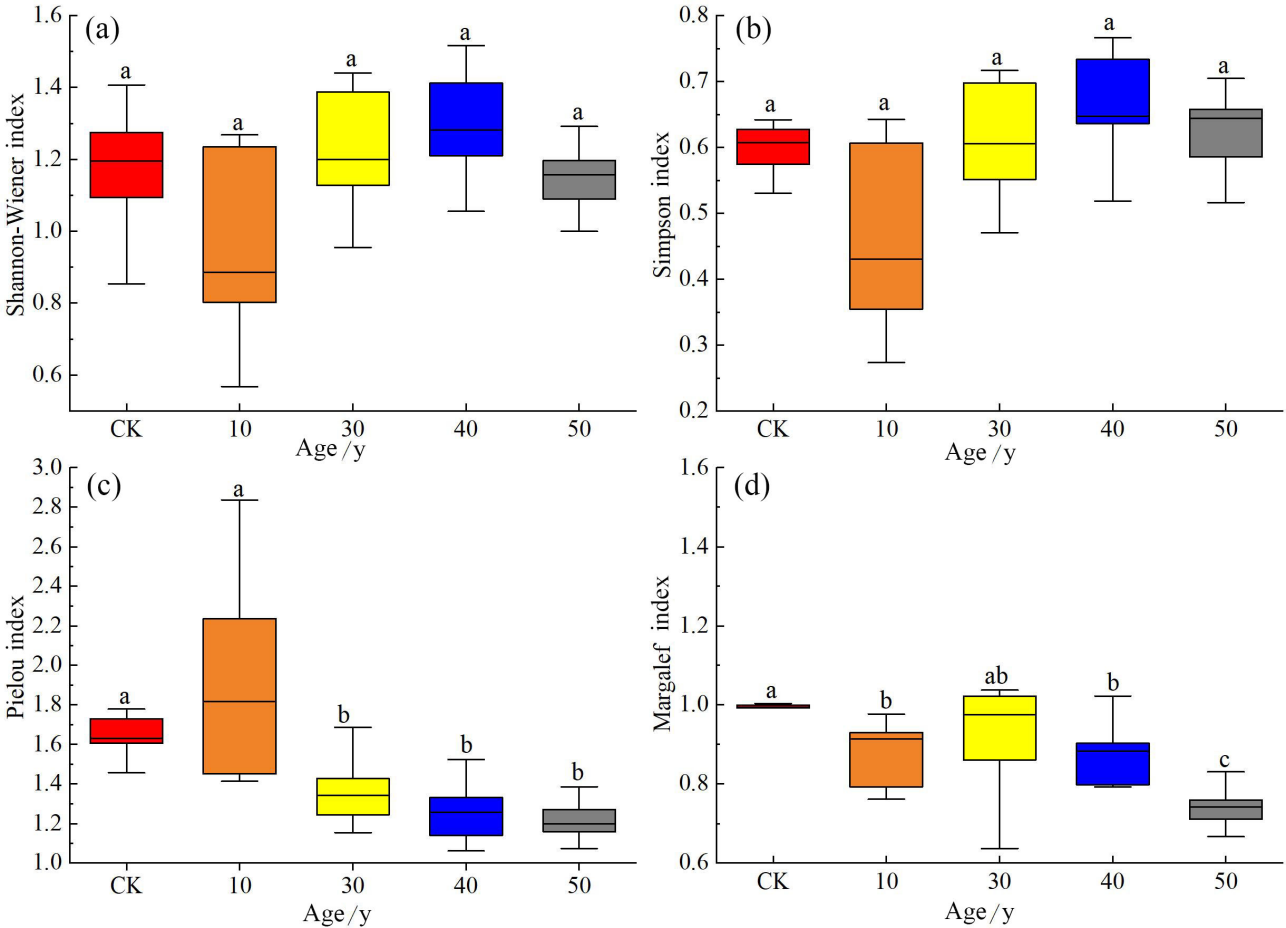
Distribution of herbaceous plant diversity indices under *Poplar* plantations of dfferent stand ages. **(a)** ShannonWiener index; **(b)** Simpson index; **(c)** Pielou index; **(d)** Margalef index. Different lowercase letters indicate significant differences among various stand ages at the 0.05 probability level.

The Shannon-Wiener index (Equation 2), and Simpson index (Equation 3), and Margalef index showed a trend of initial decrease, followed by an increase and a subsequent decrease from the CK group to the 10 y, 30 y, 40 y, and 50 y stand ages. No significant differences in the Shannon-Wiener index and Simpson index were detected among different stand ages (*p* > 0.05, **Figures 2A, B**). The Pielou index exhibited a declining trend with increasing stand age, and significant differences were detected between the CK group, 10 y stand, and the 30 y, 40 y, 50 y stands (*p* < 0.05) (**Figure 2C**) (Equation 4). The Margalef index reached the maximum value in the CK and the minimum value in the 50 y stand, and significant differences were observed between these two stand ages (*p* < 0.05, **Figure 2D**) (Equation 5).

### 3.3 Analysis of soil physical and chemical properties in *Poplar* plantations of different stand ages

No significant differences were detected in soil pH (7.96–8.22) and electrical conductivity (106.93–124.30 μs·cm^-^¹) among quadrats in *Poplar* plantations of different stand ages (**Table 3**, *p* > 0.05). Soil bulk density (SBD) decreased gradually with increasing stand age, with the maximum value observed in the CK plot (1.55 g·cm^-^³) and the minimum value (1.28 g·cm^-^³) in the 50-year-old stand. Significant differences were found between soil bulk density in the CK and that in each stand age (*p* < 0.05). Soil water content (SWC) increased gradually with stand age, reaching the maximum value (6.36%) in the 50-year-old stand and the minimum value (2.57%) in the 10-year-old stand. Soil total porosity (STP) was the highest (54.68%) in the 50-year-old stand, and significant differences were found between the 50-year-old stand and each of the other stand ages (*p* < 0.05). Soil organic carbon content (SOC) increased gradually with stand age, peaking at 9.44 g·kg^-^¹ in the 50-year-old stand and bottoming at 4.48 g·kg^-^¹ in the 10-year-old stand. Soil organic carbon content in the 50-year-old stand differed significantly from that in the CK and other stand ages (*p* < 0.05). Soil available potassium (AK) increased gradually with stand age, reaching the maximum value (93.67 mg·kg^-^¹) in the 50-year-old stand, and significant differences were detected among different stand ages (*p* < 0.05). Soil total nitrogen (TN) content increased gradually with stand age, but no significant differences were observed among different stand ages (*p* > 0.05). Soil total phosphorus (TP) content at each stand age was lower than that in the CK and showed a gradual decreasing trend with increasing stand age.

**Table 3:** Soil physicochemical properties of *Poplar* plantations of different forest ages.

Age (y)	pH	EC (μs·cm^-1^)	SBD (g·cm^-3^)	SWC (%)	STP (%)	SOC (g·kg^-1^)	AK (mg·kg^-1^)	TN (g·kg^-1^)	TP (g·kg^-1^)
CK	8.22 ± 0.10a	124.30 ± 24.49a	1.55 ± 0.04a	3.17 ± 0.25bc	46.87 ± 2.45b	5.95 ± 0.19c	68.67 ± 2.47c	0.47 ± 0.16b	0.62 ± 0.06a
10	7.96 ± 0.15a	120.28 ± 33.06a	1.44 ± 0.03ab	2.57 ± 0.52c	49.11 ± 0.37b	4.48 ± 0.06d	78.17 ± 9.00bc	0.37 ± 0.09b	0.61 ± 0.15a
30	8.05 ± 0.11a	106.27 ± 17.70a	1.43 ± 0.03ab	2.70 ± 0.22c	46.45 ± 0.45b	6.39 ± 0.20b	84.67 ± 2.84ab	0.39 ± 0.16b	0.46 ± 0.01b
40	8.07 ± 0.22a	111.07 ± 13.76a	1.37 ± 0.04ab	3.51 ± 0.08b	47.37 ± 2.84b	6.64 ± 0.23b	85.33 ± 6.53ab	0.43 ± 0.09b	0.45 ± 0.03b
50	8.06 ± 0.14a	106.93 ± 8.41a	1.28 ± 0.22b	6.36 ± 0.42a	54.68 ± 0.69a	9.44 ± 0.29a	93.67 ± 5.51a	0.54 ± 0.37a	0.45 ± 0.03b

Different lowercase letters indicate significant differences among various indices at the 0.05 probability level. EC, Electrical conductivity; SBD, Soil bulk density; SWC, Soil water content; STP, Soil total porosity; SOC, Soil organic carbon; AK, Available potassium; TN, Total nitrogen; TP, Total phosphorus. The same applies to the following tables and figures.

### 3.4 Coupling relationship between plant diversity and environmental factors under *Poplar* plantations of different stand ages

#### 3.4.1 Correlation analysis of understory plant diversity with stand factors and soil factors

**Figure 3:**
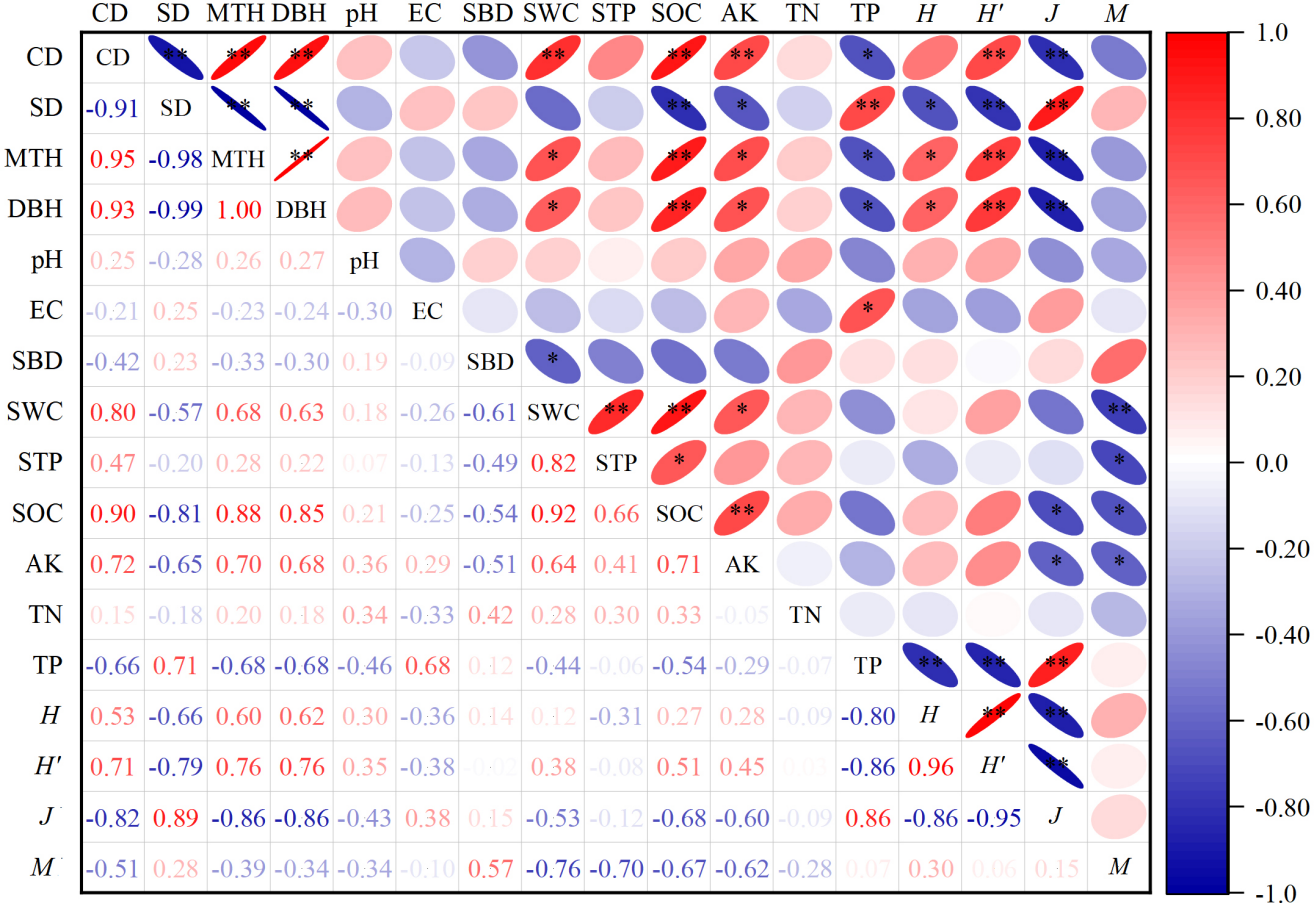
Correlation analysis between plant diversity and environmental factors. “*”: *p*<0.05; “**”: *p*<0.01; CD, canopy closure; SD, stand density; MTH, mean tree height; DBH, diameter at breast height; EC, electrical conductivity; SBD, soil bulk density; SWC, soil water content; STP, soil total porosity; SOC, soil organic carbon; AK, available potassium; TN, total nitrogen; TP, total phosphorus; *H*, Shannon-Wiener index; *H’*, Simpson index; *J*, Pielou index; M, Margalef index. The same notations are adopted in the following tables and figures.

Results indicated that a significantly negative correlation was detected between the diversity index *H* and SD (*p*<0.05), while significantly positive correlations were determined between the diversity index *H* and MTH as well as DBH (*p*<0.05), and an extremely significantly negative correlation was observed between the diversity index *H* and TP (*p*<0.01). Extremely significantly positive correlations were identified between the dominance index *H’* and CD, MTH as well as DBH (*p*<0.01), and extremely significantly negative correlations were found between the dominance index *H’* and SD as well as TP (*p*<0.01). Extremely significantly negative correlations were measured between the evenness index *J* and CD, MTH as well as SBD (*p*<0.01), whereas extremely significantly positive correlations were recorded between the evenness index J and SD as well as TP (*p*<0.01), and significantly negative correlations were detected between the evenness index J and SOC as well as AK (*p*<0.05). An extremely significantly negative correlation was observed between the richness index M and SWC (*p*<0.01), and significantly negative correlations were determined between the richness index M and STP, SOC as well as AK (*p*<0.05). No significant correlations were observed between pH, EC, SBD, TN and all plant diversity indices (*p*>0.05) (**Figure 3**).

#### 3.4.2 Multiple regression analysis of understory plant diversity with stand factors and soil factors

Results of multiple regression analysis applied to plant diversity indices, stand factors and soil factors demonstrated that the *R*^2^ values for the Shannon index, Simpson index, Pielou index and Margalef index were all higher than 0.73. Strong explanatory power of the selected environmental factors on the understory plant diversity pattern was confirmed with satisfactory fitting performance (**Table 4**). The primary influencing factors for the Shannon-Wiener index were identified as CD, SD and SWC, among which SWC exerted positive effects while CD and SD exerted negative effects. The dominant influence factor for the Simpson index was determined to be SWC with positive effects. The main influencing factors for the Pielou index were detected as CD and SD with positive effects. The key influencing factors for the Margalef index were recognized as SWC and SOC, among which SOC presented positive effects and SWC presented negative effects. On the basis of the above results, CD, SD, SWC and SOC were regarded as the major environmental factors regulating understory plant diversity.

**Table 4:** Multiple regression equations of understory plant diversity with stand factors and soil factors.

Diversity index	Multiple regression equation	*R* ^2^
Shannon index	*H* = 0.323 + 0.099MTH-0.100DBH-0.888CD*-0.001SD*+0.323pH+1.301SBD+0.182SWC*-0.039STP-0.008AK-0.865TN	0.774
Simpson index	*H’* = -0.596 + 0.060MTH-0.052DBH-0.700CD+0.204pH+0.807SBD+0.126SWC*-0.018STP-0.006AK-0.527TN	0.733
Pielou index	*J* = 2.212-0.163MTH+0.170DBH+3.872CD*+0.005SD*-1.012pH-0.009EC-1.045SBD-0.656SWC+0.035STP+0.297SOC+0.032AK+1.102TN	0.943
Margalef index	*M* = -0.540 + 0.062MTH0.056DBH-0.161pH-0.0028EC+1.370SBD-0.142SWC*+0.213SOC*-0.776TN	0.789

“*”: *p*<0.05; “**”: *p*<0.01; CD: canopy closure; SD, stand density; MTH, mean tree height; DBH, diameter at breast height; EC, electrical conductivity; SBD, soil bulk density; SWC, soil water content; STP, soil total porosity; SOC, soil organic carbon; AK, available potassium; TN, total nitrogen; TP, total phosphorus; *H*, Shannon-Wiener index; *H’*, Simpson index; *J*, Pielou index; M, Margalef index. The same notations are adopted in the following tables and figures.

## 4 Discussion

### 4.1 Distribution patterns of plant diversity under *Poplar* plantations of different stand ages

Prior research has verified that the dynamic distribution patterns of understory plant diversity in artificial forests vary significantly across different stand developmental stages, and such variations are predominantly regulated by stand age, geographical background and ambient environmental conditions (Ford et al., 2000; Barsotti et al., 2026). The present study found that the overall diversity of understory herbaceous plants, as reflected by the Shannon-Wiener and Simpson indices, followed a trend of initial increase and subsequent decrease from the control grassland to 50-year-old *Poplar* plantations. Plant diversity levels peaked in 40-year-old stands and experienced a mild reduction in 50-year-old stands. Such phenomena suggest that middle-aged plantation stands can create favorable understory habitats with optimal light conditions, water availability and soil nutrient status. These superior microenvironmental conditions are capable of sustaining higher levels of understory plant diversity (Shi et al., 2021; Wen et al., 2025). The above variations can be explained by two core mechanisms. The first is the threshold effect derived from canopy closure and light resource restriction. At the early stage including grassland and young forest stands, incomplete canopy closure ensures sufficient understory light. A large number of drought tolerant and heliophytic herbaceous species such as Setaria viridis, Humulus scandens and Chromolaena odorata achieve rapid growth and colonization, which promotes the rapid rise of plant diversity. Gradual canopy development forms heterogeneous light environments composed of canopy gaps and shaded understory zones. Such habitat conditions maintain the survival of heliophytes and provide colonization opportunities for shade tolerant species, thereby sustaining relatively high plant diversity. In stands ranging from forty to fifty years old, canopy density exceeds the critical threshold value. Understory light intensity declines sharply and fails to satisfy the photosynthetic demands of most herbaceous plants. This shift transforms the dominant influencing factor of understory herb communities from light competition to light restriction. Light sensitive species gradually disappear from local communities and plant diversity declines accordingly. The second mechanism refers to physical and chemical inhibition effects caused by dead branches and litters. Mature *Poplar* stands aged forty to fifty years accumulate abundant dead branches and fallen leaves to form thick litter layers. Such physical barriers prevent seeds from making effective contact with soil and hinder the normal emergence of plant seedlings. Existing research has proven that litter accumulation presents significantly negative correlations with herbaceous coverage and species richness (Loydi et al., 2013). In terms of allelopathic effects, decomposing *Poplar* leaves release secondary metabolites dominated by phenolic acids. The accumulation of these substances in soil restrains seed germination and seedling growth of other plant species and exerts strong inhibitory effects on herbaceous plants. Older forest stands show more prominent accumulation effects of such allelochemical substances and further narrow the survival niches of understory herbaceous plants. Certain existing studies have yielded findings inconsistent with the results of the current investigation. Relevant observations on Eucalyptus plantations have illustrated that understory plant diversity declines initially and rises gradually with the extension of stand age (Li et al., 2023). Such inconsistent ecological patterns can be explained by the intensive allelopathic effects and strong water competition ability of Eucalyptus trees, as well as the heterogeneity of regional soil environmental conditions (Qin et al., 2018; Chen et al., 2024). The variation trends of plant diversity across differently aged artificial forests are therefore highly specific to tree species and regional habitat backgrounds. Stand developmental processes also alter forest canopy structure, which serves as another critical driver for the temporal variation of understory plant diversity. The present study detected continuous growth in canopy closure along with the aging of *Poplar* stands. Dense canopy coverage substantially reduces understory light transmittance and constrains the growth of heliophytic herbaceous plants. This ecological process primarily accounts for the gradual reduction of the Pielou index in aging *Poplar* plantations. Light availability consequently acts as a major limiting resource for understory community development. Species with superior adaptability to low light conditions gain greater competitive advantages, which strengthens the dominance of superior species, simplifies community structure, and ultimately reduces the overall evenness of understory plant assemblages (Barbier et al., 2008).

Resource competition and niche differentiation serve as essential mechanisms shaping the spatiotemporal variations of plant diversity across forest stands at different developmental stages. The current study showed that the Margalef index reached its maximum value in 30-year-old *Poplar* stands with a canopy closure of 0.46 and a stand density of 450 trees per hectare, followed by a gradual decline in older stands. This variation pattern arises from the combined effects of soil nutrient and water competition, niche differentiation and competitive exclusion among understory plant species. During the early and middle growth stages of *Poplar* plantations, heterogeneous understory microhabitats formed by uneven light transmission and litter distribution support the coexistence of diverse herbaceous species (Landuyt et al., 2019). Further stand maturation enhances the capacity of *Poplar* root systems to occupy soil space and absorb soil resources. The continuous reduction of understory light availability narrows the niche breadth of herbaceous communities and eliminates species with weak competitive adaptability (Matsushita et al., 2025). The *Poplar* plantations sampled in this research are monoculture stands characterized by simplified community composition, with understory layers dominated entirely by herbaceous vegetation and a complete absence of shrub species. Previous studies have demonstrated that mixed forests outperform monoculture plantations in canopy structural complexity and soil nutrient status through interspecific complementarity. Such structural and functional improvements generate more heterogeneous microhabitats that promote the colonization and growth of both shrub and herbaceous species (Jactel et al., 2018; Kremer et al., 2025). Accordingly, the conversion of pure *Poplar* plantations to mixed forest stands represents a feasible strategy to enhance ecosystem stability and improve biodiversity maintenance capacity of artificial forests in the Luxi Yellow River Floodplain.

### 4.2 The distribution pattern of physical and chemical properties of soil under artificial forests of *Poplar* trees of different ages

This study confirms that stand age acts as a key driving factor altering soil bulk density, soil water content and total phosphorus content in *Poplar* plantation ecosystems. Soil bulk density presents a pronounced decreasing trend in stands older than 40 years, which implies that the amelioration of soil structural properties via vegetation restoration gradually intensifies alongside stand maturation. Higher levels of soil water content and total soil porosity are observed in 50-year-old stands relative to other age groups. This phenomenon can be explained by thickened surface litter layers, improved soil water retention capability and long-term nutrient accumulation in mature plantation ecosystems. These changes reflect the recovery potential of soil water and phosphorus reservoirs during the late developmental stage of artificial forests (Hayashi and Mizuyama, 2006). Soil pH values remain stable across all stand age groups due to the robust soil buffering capacity of the study area. Although stand aging promotes litter input, organic acid exudation and root respiratory activities, such ecological changes generate limited effects on mitigating regional soil salinization (Zhu et al., 2024). These results align with previous investigations on Sonneratia apetala and Castanopsis hystrix plantations with different stand ages (Jiang et al., 2023; Zhang, 2023). Such consistence indicate that the regulatory effects of stand age on soil pH can be masked by regional environmental backgrounds including soil parent materials and local climatic conditions in specific forest ecosystems. Soil electrical conductivity shows relatively low values in 50-year-old stands and presents unstable fluctuations in younger stands. The well-developed root systems and high transpiration intensity of mature stands may facilitate the absorption and upward migration of soluble soil salts. Meanwhile, litter layers can intercept and redistribute precipitation, thereby modulating soil salt leaching processes (Peters et al., 2021). The variations of soil electrical conductivity along the stand age gradient therefore derive from complex interactive processes among hydrological dynamics, vegetation physiological activities and inherent soil physicochemical characteristics.

The concentrations of soil organic carbon and total nitrogen peaked in 50-year-old *Poplar* stands. The accumulation of these soil nutrients can be largely explained by the increased litter input and altered litter quality during stand maturation, such as elevated lignin concentrations that facilitate the stable sequestration of soil organic matter (Cotrufo et al., 2013). Improved symbiotic interactions between vegetation roots and soil microorganisms further contribute to efficient nitrogen fixation and nutrient retention in aging plantation soils (Midgley and Phillips, 2016). In contrast, soil total phosphorus content declined continuously along the stand age gradient and gradually stabilized in the oldest stands. This changing trend differs from the findings of several previous studies that recorded phosphorus accumulation in aging forests. Such inconsistency is likely controlled by low phosphorus levels in local soil parent materials and biological immobilization processes. Phosphorus tends to be fixed by iron and aluminum oxides in soils with acidic to neutral pH conditions, which further reduces its bioavailability. As a fast-growing tree species, *Poplar* consumes substantial soil phosphorus resources for growth metabolism. Long-term continuous nutrient uptake by vegetation gradually depletes soil phosphorus reserves in plantation ecosystems (Pan et al., 2024). Given the prevalent phosphorus deficiency in the Luxi Yellow River Floodplain, targeted field management measures including combined application of phosphorus and organic fertilizers are recommended to mitigate phosphorus limitation. Such regulation strategies can support healthy stand growth and promote the long-term sustainable development of local forest ecosystems.

### 4.3 Coupling relationship between environmental factors and understory plant diversity

This study found that soil pH and electrical conductivity showed no significant correlations with any plant diversity metrics. Such results indicate that soil pH and salinity levels across the study area remain within the adaptive tolerance range of local understory plant communities, and these soil properties do not function as core environmental constraints on the distribution of herbaceous vegetation. Soil bulk density also exhibited insignificant correlations with diversity indices. As a stable soil physical attribute that reflects compaction status, soil bulk density presents limited temporal variation across stand age gradients. Its influences on understory plant diversity are predominantly indirect, mediated through alterations in water infiltration efficiency, root expansion space and soil microbial activities (Richardson et al., 2009). The overall concentrations of total nitrogen and total phosphorus in local *Poplar* plantations were relatively low in this research. The absence of significant correlations between total nitrogen and plant diversity implies that vegetation development is subject to combined nitrogen and phosphorus limitation, among which phosphorus scarcity acts as the primary restrictive factor (Elser et al., 2007). Herbaceous plant growth displays higher sensitivity to fluctuations in soil phosphorus availability, making total phosphorus rather than total nitrogen the dominant nutrient driver shaping understory diversity patterns. Multiple regression analysis revealed that the Shannon diversity index was significantly associated with canopy density, stand density and soil water content. Increased canopy coverage and stand density reduce light transmittance to the forest floor, suppress the growth of heliophytic herbaceous species, and consequently lower overall plant species diversity. This finding supports the classical light competition theory and confirms light availability as the primary limiting resource for understory plant community development. The Simpson index presented a significant positive correlation with soil water content, which suggests that improved soil moisture conditions facilitate the growth of dominant species and enhance community competitive dominance. Frequent seasonal drought in the Yellow River Floodplain renders water availability a critical determinant of species competitive superiority and community construction (Sebastià et al., 2005; Chelli et al., 2024). The Pielou index was positively correlated with canopy density and stand density. Dense stand structures intensify interspecific competition among arbor layers for light, water and soil nutrients, which restricts the resource acquisition and growth space of understory vegetation. Intense environmental filtering eliminates most vulnerable plant species and only allows the survival of a small number of shade tolerant species with evenly distributed individual abundance. This structural simplification of plant communities under harsh microhabitat conditions contributes to improved community evenness, which aligns with the general mechanism of environmental filtering (Wang et al., 2022). The Margalef index showed a significant negative correlation with soil water content and a significant positive correlation with soil organic carbon. High soil moisture promotes the vigorous growth of *Poplar* trees, which aggravates belowground root competition and reduces understory light penetration, ultimately decreasing herbaceous species richness. In contrast, elevated soil organic carbon improves overall soil fertility and provides favorable conditions for the colonization and survival of diverse plant species. In the Yellow River Floodplain, sufficient water resources substantially strengthen the competitive advantages of *Poplar* trees and inhibit herbaceous community development, whereas improved soil nutrient conditions effectively sustain higher plant diversity (Pan et al., 2025). These contrasting responses reflect the distinct trade off effects between soil water and nutrient resources in regulating understory community characteristics. Combined correlation and multiple regression analyses further clarified the comprehensive interactive relationships between plant diversity and multi-dimensional environmental variables. The established statistical model achieved reliable fitting performance and verified that stand structural characteristics impose significant indirect effects on understory plant diversity through modulating soil physicochemical properties including soil organic carbon. These outcomes demonstrate the cascading regulatory relationships among forest stand structure, soil environmental conditions and understory plant community assembly. In conclusion, canopy density, stand density, soil water content and soil organic carbon constitute the key environmental factors driving the temporal variation of understory plant diversity in differently aged *Poplar* plantations within the study area. Light availability serves as the dominant regulator of plant species diversity and community evenness. The optimization of stand structural attributes such as stand density and vegetation composition can effectively improve soil water and nutrient conditions, thereby enhance understory plant diversity and maintain the ecological stability of artificial forest ecosystems in the Yellow River Floodplain.

This study primarily explored the responses of understory plant diversity to stand age, stand structure and soil physicochemical properties, while topographic variables such as altitude and slope were not incorporated into the analytical framework. Topographic conditions can reshape the spatial distribution of plant diversity by regulating the redistribution of hydrothermal resources and soil developmental processes (Matsushita et al., 2025). In addition, field sampling in the present research was conducted only once during the growing season, which fails to capture seasonal fluctuations and interannual variability of understory herbaceous plant communities. Future investigations can implement long term multi seasonal field monitoring and integrate multidimensional influencing factors including topographic features, soil microbial properties and litter functional traits. Such comprehensive approaches will help elaborate the underlying spatiotemporal maintenance mechanisms of plant diversity in artificial plantation ecosystems with higher accuracy and systematicity.

## 5 Conclusion

This study systematically surveyed understory plant communities across *Poplar* plantations with different stand ages in the Luxi Yellow River Floodplain. A total of 21 vascular plant species were identified, which belonged to 21 genera and nine families. The understory communities were predominated by *Setaria viridis*, *Phragmites australis* and *Erigeron canadensis*. The control grassland group exhibited the highest species richness with a total of 12 recorded species. The Shannon-Wiener and Simpson diversity indices of herbaceous communities presented a consistent trend of initial increase and subsequent decline along the stand age gradient, although the differences among age groups were not statistically significant. The Pielou index peaked in 10-year-old stands and declined continuously with stand maturation. The Margalef index showed minor fluctuations across all sampling sites, reaching the highest level in 30-year-old stands before undergoing a slow decreasing trend. Soil pH, electrical conductivity and total nitrogen content did not vary significantly among *Poplar* stands of different ages. Soil bulk density and total phosphorus content decreased gradually as stand age increased, with the minimum values of 1.28 g·cm^-^³ and 0.45 g·kg^-^¹ observed in 50-year-old stands. Conversely, soil water content, total soil porosity, soil organic carbon, available potassium and total nitrogen increased progressively with stand age and attained their maximum values of 6.36%, 54.68%, 9.44 g·kg^-^¹, 93.67 mg·kg^-^¹ and 0.54 g·kg^-^¹ respectively in the oldest stands. Correlation analysis and multiple regression analysis collectively demonstrated that canopy density, stand density, soil water content and soil organic carbon function as the core environmental factors driving the dynamic variations in understory plant diversity of *Poplar* plantations in the Luxi Yellow River Floodplain.

## Data Availability

The original contributions presented in the study are included in the article/supplementary material. Further inquiries can be directed to the corresponding author.
